# Effect of 4DryField® PH on blood loss in hip bipolar hemiarthroplasty following intracapsular femoral neck fracture – a randomized clinical trial

**DOI:** 10.1186/s12891-021-03983-6

**Published:** 2021-01-26

**Authors:** Benjamin Lucas, Christian Riebau, Juliane Mohr, Gerald Pliske, Felix Walcher, Stefan Piatek

**Affiliations:** Department of Trauma Surgery, Otto-von-Guericke University Magdeburg, Leipziger Str. 44, D-39120 Magdeburg, Germany

**Keywords:** 4DryField® PH, Blood loss, Femoral neck fractures, Hip arthroplasty

## Abstract

**Background:**

One of the most common complications of hip arthroplasty is excessive blood loss that could necessitate allogenic blood transfusion, which is further associated with other complications, such as infections, transfusion reactions or immunomodulation. In gynecology, 4DryField®PH, an absorbable polysaccharide-based formulation, is used for hemostasis and adhesion prophylaxis. In this study, we evaluated its hemostatic effect in patients undergoing hip bipolar hemiarthroplasty following intracapsular femoral neck fracture.

**Methods:**

We studied 40 patients with intracapsular femoral neck fractures (Garden III or IV) admitted at our institution between July 2016 and November 2017. We included patients above 60 years with simple fracture and without pathologic fractures. Patients were randomized into intervention and control groups. The intervention group received 5 g of 4DryField® PH (subfascially and subcutaneously) during wound closure. Three drainages were inserted in a standardized manner (submuscular, subfascial, and subcutaneous) and drainage volume was measured immediately before extraction. Total blood loss was calculated using Mercuriali’s formula and standard hemograms upon admission and five days after surgery. Volume of postoperative hematoma was measured using point-of-care ultrasound seven days after surgery.

**Results:**

Volume of the postoperative hematoma was reduced by 43.0 mL. However, significant reduction of total blood loss and drainage volume was not observed.

**Conclusions:**

We observed that 4DryField® PH had a local hemostatic effect, thereby reducing volume of the postoperative hematoma. However, this reduction was small and had no effect on the total blood loss. Further studies are warranted to improve the application algorithm.

**Trial registration:**

DRKS, DRKS00017452, Registered 11 June 2019 – Retrospectively registered.

## Background

Femoral neck fractures are common in elderly patients [1]. Fractures with or without minimal displacement (Garden I–II) are managed with hip reconstruction (dynamic hip screw). On the other hand, displaced fractures (Garden III–IV) require arthroplasty [1].

One of the most common complications of bipolar hip arthroplasty for femoral neck fractures is the excessive intra- and postoperative blood loss that could necessitate allogenic blood transfusion [1], which is further associated with other complications, such as infections and transfusion reactions [2]. Moreover, Goubran et al. demonstrated that blood transfusions have an immunomodulatory effect, which may result in immunosuppression [3]. Given this context, it is prudent to minimize intra- and postoperative blood loss.

In addition to optimized surgical procedures, several methods have been evaluated to decrease blood loss, such as application of tranexamic acid, hypotensive anesthesia, various blood salvage techniques, and pharmacologic approaches [1, 4]. One such method is the application of 4DryField® PH (PlantTec Medical, Lüneburg, Germany), an absorbable polysaccharide-based formulation [5, 6], which acts by binding to the fluid component of blood, thereby concentrating blood cells and proteins. This accelerates clotting and contributes to hemostasis. This formulation is useful in adhesion prophylaxis as well. In a few days following application, 4DryField® PH is processed by endogenous amylases and resorbed [7]. In literature, an immediate hemostatic effect of 4DryField® PH after resection of endometriosis has been reported [6].

In this study, we evaluated the effect of 4DryField® PH on postoperative blood loss in patients undergoing hip bipolar hemiarthroplasty following intracapsular femoral neck fracture. In light of the described hemostatic effects, we expected to observe a distinct decrease in total blood loss calculated using the Mercuriali’s formula [8, 9], based on the patient’s blood volume calculated using the Nadler formula [9, 10].

## Methods

### Ethical approval and consent to participate

This study was conducted in accordance with the ethical principles outlined in the Declaration of Helsinki. The study protocol was approved by the local research ethics board (REB#: 08/16). Prior to inclusion in the study, written informed consent was obtained from all participants or their legal representatives (in case of incapability) after they were explained details about the standardized surgical procedure, 4DryField® PH, randomization procedure, and postoperative assessments. The trial was retrospectively registered on 11/06/2019 in the German Clinical Trials Register (registration number: DRKS00017452).

### 4DryField® PH

4DryField® PH (PlantTec Medical, Lüneburg, Germany) is an absorbable polysaccharide-based formulation and is applied directly to the surgical site as a powder. The formulation is hypoallergenic and is prepared from potato starch [5–7].

### Study design and 4DryField® PH application

**Fig. 1:**
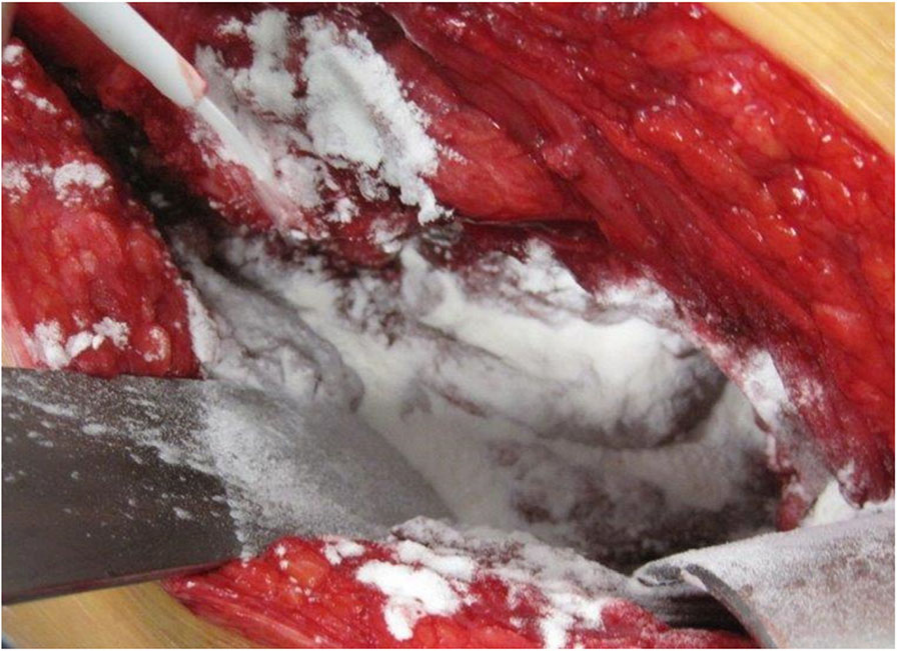
Application of 4DryField® PH. In the 4DryField® group, 5 g of the polysaccharide powder was applied subfascially and subcutaneously in equal proportion

**Fig. 2:**
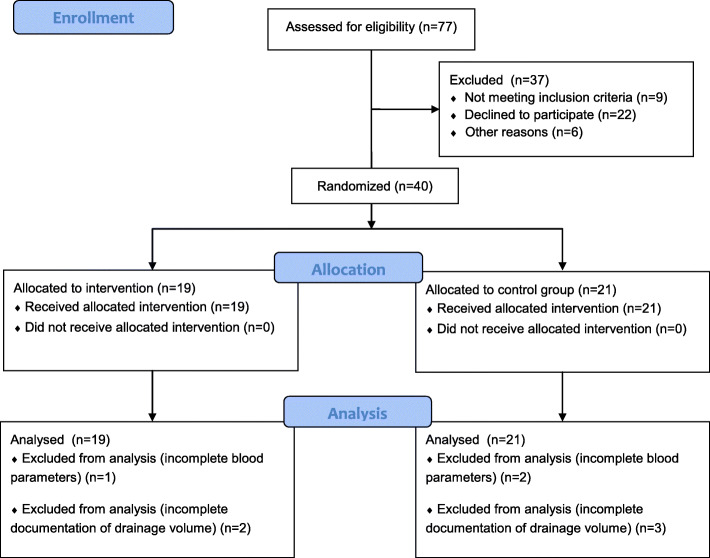
CONSORT flow diagram. In this study we assessed for eligibility 77 patients. As 37 patients were excluded (*n* = 9 not meet inclusion criteria, *n* = 22 declined to participate and *n*=6 for other reasons) we randomized 19 patients in intervention and 21 in control group. Due to incomplete blood parameters 1 patient in intervention and 2 patients in control group were excluded in analysis of total blood loss. Due to incomplete documentation in drainage volume 2 patients in intervention and 3 patients in control group were excluded from analysis

We studied 40 patients with intracapsular femoral neck fractures admitted at our institution between July 2016 and November 2017. We only included patients above 60 years of age with simple fracture and without pathologic fracture. For comparison of the comorbidities, we used the Charlson Comorbidity Index [11]. After obtaining informed consent to participate in this study, patients were randomly allocated to the intervention group (19 patients), receiving 4DryField® PH, and the control group (21 patients). The procedure of hip bipolar hemiarthroplasty was standardized based on a modified approach described by Bauer [12]. The operations were conducted by 6 different surgeons. The size of the implant (Peter Brehm GmbH, Weisendorf, Germany) was determined intraoperatively based on the diameter of the femoral head, fitting of the stem in x-ray, and leg length. The femoral stems were cemented. In the intervention group, we applied 5 g of 4DryField® PH subfascially and subcutaneously, in equal proportion during wound closure (Fig. 1). Tranexamic acid was neither topical nor intravenously administered. Relating to the standard surgical procedure of our clinic, we inserted three Redon (Primed Halberstadt Medizintechnik GmbH, Halberstadt, Germany) drainages (submuscular, subfascial and subcutaneous) in all patients of both groups. The postoperative prophylaxis of venous thromboembolism was performed with low molecular weight heparin by default or with unfractionated heparin in patients with chronic kidney disease with an estimated glomerular filtration rate below 30 mL/min/1.73 m². The methodology to report this study adheres to the Consolidated Standards of Reporting Trials statement (CONSORT; Fig. 2).

### Outcome variables and measurement

For primary outcome measurement, we used the total blood loss calculated using the Mercuriali’s formula [8, 9], based on the patient’s blood volume calculated using the Nadler formula [9, 10]. To this end, we performed a hemogram on the first and fifth day after surgery. In this regard, total blood loss estimated the intra- and post-operative blood loss. To distinguish between post-operative and intra-operative blood loss, we used the drainage volume and the size of the postoperative hematoma as secondary outcome variables. Therefore, we measured the contents of the Redon drainage immediately before extraction, which was performed between the first and fourth day after surgery, depending on the daily flow rate. The volume of the postoperative hematoma at the surgical site was analyzed using ultrasound with a point-of-care device SonoSite M-Turbo (FUJIFILM SonoSite Europe, Amsterdam, Netherlands) on the seventh postoperative day.

### Randomization procedure and statistical analyses

Randomization was performed using opaque sealed envelopes. The randomization result was only known by the surgeon who operated on the patient.

Data was analyzed using Statistical Package for the Social Sciences, version 24 (IBM Corp., Armonk, NY, USA). We estimated that a sample of 70 patients was needed to achieve an 80 % power, at an alpha of 0.05, to detect a decrease in total blood loss from 373 ml in the control group to 261 ml in the intervention group, with a common standard deviation of 163 ml in both groups. Considering subject attrition, a sample size of 80 patients was assumed and an interims analysis was planned after inclusion of 40 patients. All data are presented as mean ± standard deviation (SD), except for the point-of-care ultrasound of the postoperative hematoma and the Charlson comorbidity index scores. These were presented as median (interquartile range; IQR) because of the non-normal distribution of the data. Normality of distributions was tested using the Kolmogorov-Smirnov test. Comparison of the two groups in terms of total blood loss and drainage volume was done using a t-test. On the other hand, we used the Mann-Whitney U-test to compare volumes of postoperative hematomas and the Charlson comorbidity index scores because of the non-normal distribution of the data (Kolmogorov-Smirnov, *P* < 0.001). *P* values lower than 0.05 were considered statistically significant.

## Results

After randomization of 40 patients (Fig. 2) an interim analysis was conducted wherein we studied 19 patients in the intervention group and 21 patients in the control group. The intervention group comprised of 4 men and 15 women with a mean age of 82 years. The control group consisted of 7 men and 14 women with a mean age of 80 years (Table 1). The Charlson comorbidity index scores of both groups were not significantly different (4DryField® PH group 6 [IQR: 1] vs. control group 5 [IQR: 3]; Mann-Whitney U-test *p* = 0.872). The perioperative anticoagulation is comparable between the two groups (Table 2). Depending on the patient’s renal function, warfarin or new oral anticoagulants were replaced with unfractionated heparin or Low-molecular-weight heparin.

**Table 1:** Baseline demographic and clinical characteristics

	4DryField® PH	control
male	4	7
female	15	14
age (years)	82.0 +/- 9.7	80.0 +/- 6.2
CCI	6 (IQR: 1)	5 (IQR: 3)
BMI	25.5 +/- 3.8	25.8 +/- 3.6

**Table 2:** Perioperative anticoagulation

	4DryField® PH	control
anti-platelet agents	10	8
Low-molecular-weight heparin (prophylactic treatment)	15	12
Low-molecular-weight heparin (therapeutic treatment)	1	1
unfractionated heparin (prophylactic treatment)	1	7
unfractionated heparin (therapeutic treatment)	2	1

### Total blood loss

**Fig. 3:**
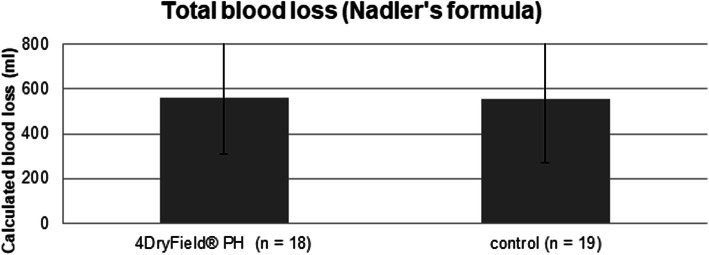
Total blood loss in the intervention and control groups. Total blood loss (calculated using Mercuriali’s formula and standard hemograms) at admission and five days after surgery. Mean total blood loss was 559.99 ± 248.26 mL in the 4DryField® PH group and 557.25 ± 284.46 mL in the control group. A significant difference in blood loss was not observed. Kolmogorov-Smirnov,*P* = 0.200; t-test, *P* = 0.971

Total blood loss was calculated using the results of the hemogram performed on admission and on the fifth day after surgery, as well as transfused erythrocyte concentrates, if applicable. Incomplete blood parameters in one patient of the intervention group and two patients of the control group resulted in their exclusion from this analysis. The data of the remaining 18 and 19 patients from the intervention and control group, respectively, were analyzed. We observed no statistically significant difference (559.99 ± 248.26 mL vs. 557.25 ± 284.46 mL) in blood loss in the 4DryField® PH group compared to the control group (Fig. 3).

### Total drainage volume

**Fig. 4:**
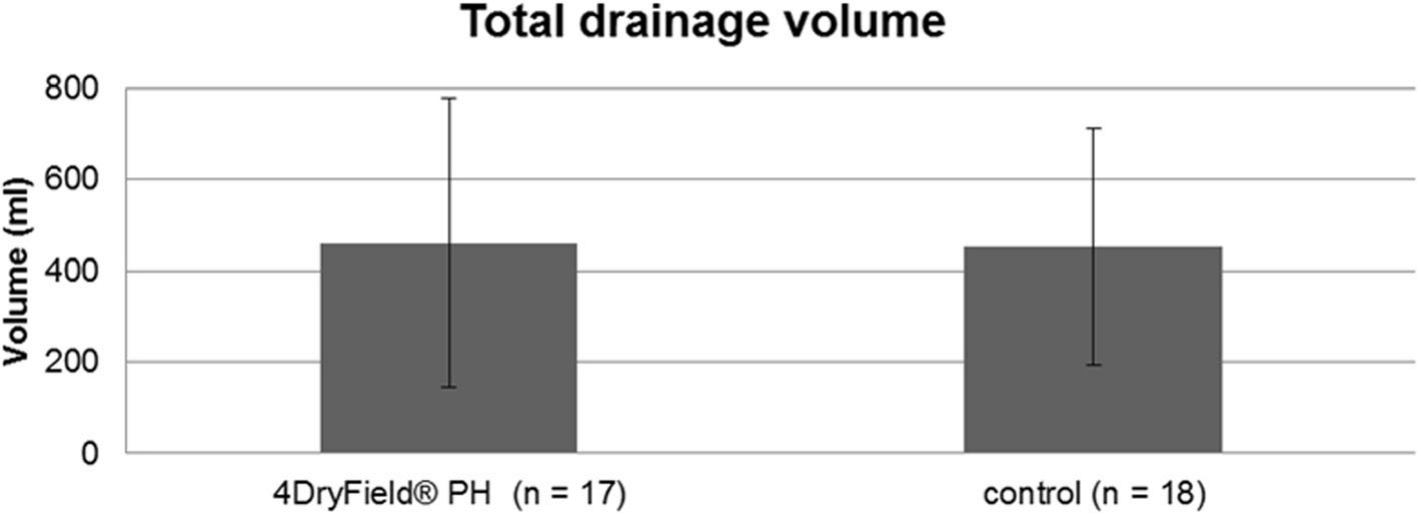
Total drainage volume. The drainage volume was measured immediately before removal. The summation of the volumes of all three drains was obtained. The drainage volume in the 4DryField® PH group (460.88 ± 297.24 mL) showed no significant difference compared to the control group (452.50 ± 224.15 mL). Kolmogorov-Smirnov, *P* = 0.063; t-test, *P* = 0.925

Incomplete documentation of drainage volume in two patients of the intervention group and 3 patients of the control group resulted in their exclusion from this analysis. Therefore, the data of the remaining 17 and 18 patients from the intervention and control, respectively, were analyzed. In contrast to total blood loss, a slight increase in total drainage volume was seen in the 4DryField® PH group (460.88 ± 297.24 mL vs. 452.50 ± 224.15 mL) compared to the control group, however, this difference was not statistically significant (Fig. 4).

### Volume of postoperative hematoma

**Fig. 5:**
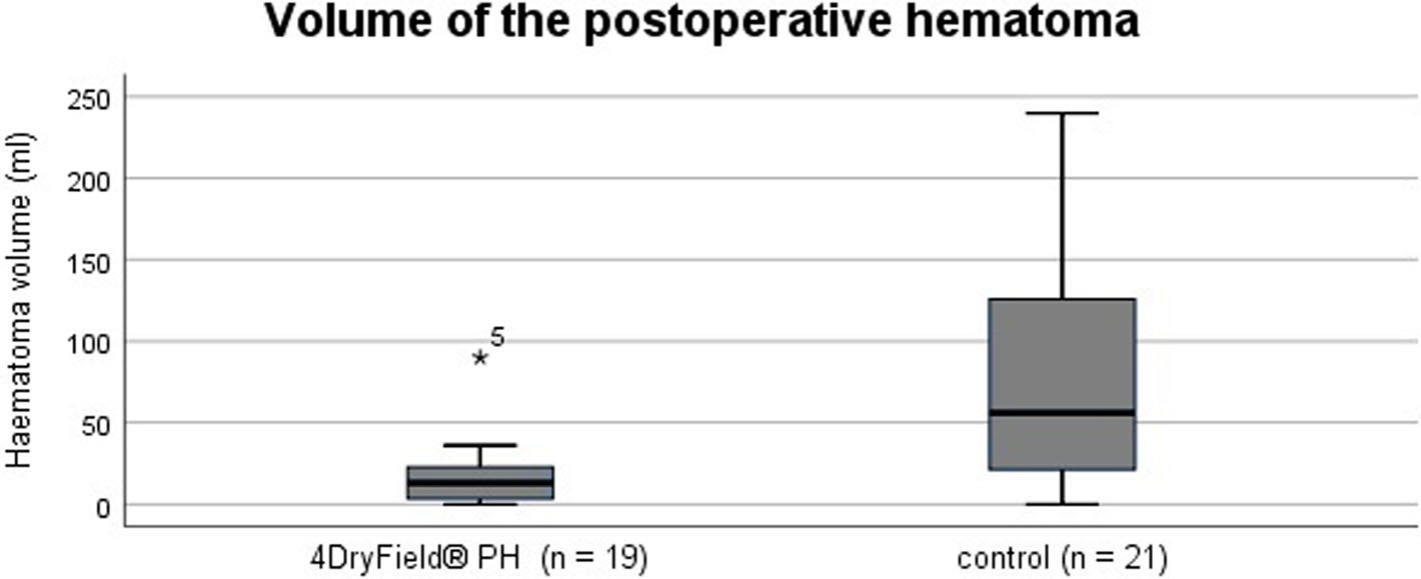
Volume of the postoperative hematoma. The volume of the postoperative hematoma at the surgical site was measured using point-of-care ultrasound seven days after surgery. A significant reduction was observed in the 4DryField® PH group (13.0 (24.0) vs 56.0 (118.0) mL). Kolmogorov-Smirnov, *P* < 0.001; Mann-Whitney U-test, *P* = 0.003

Sonography data of all 19 patients in the intervention group and 21 patients in the control group were available. The volume of postoperative hematoma was significantly lower in the 4DryField® PH group (13.0 [24.0] mL vs. 56.0 [118.0] mL; *P* < 0.05) (Fig. 5) compared to the control group. In the control group, 7 patients presented with a hematoma size of above 100 ml. Moreover, two of these patients needed surgical revision, as mentioned below. In the intervention group, all patients had a hematoma size of below 100 ml. Surgical revisions were not required in the intervention group because the postoperative hematomas were small.

### Monitoring of complications

One patient in the intervention group developed a distal periprosthetic fracture, needing surgical revision five days after the initial surgery, while one patient in the control group had luxation of the hip bipolar prosthesis on the eighth postoperative day. Surgical revision of the postoperative hematoma was done in two patients in control group on the sixth and tenth postoperative days, respectively.

## Discussion

We found that the use of 4DryField® PH was associated with reduced volume of postoperative hematoma in patients of hip bipolar hemiarthroplasty following intracapsular femoral neck fracture. However, its effect on total blood loss and total drainage volume was not remarkable. Since the difference in total blood loss between the intervention and control group was much less than what we expected, we decided to stop the trial.

Total blood loss in hip arthroplasty includes blood loss intra- and postoperatively. Several strategies have been used to minimize blood loss and to avoid blood transfusion, which may cause adverse effects, such as infection and transfusion reactions [1, 3]. In this regard, pre- and postoperative administration of tranexamic acid has been effective [13–15]. However, its use is contraindicated in patients with a history of thromboembolism, and is associated with adverse events, such as allergies [16]. In cases of arthroplasty, tranexamic acid does not significantly increase the rate of deep venous thrombosis or pulmonary embolism. [4, 15, 17]. However, there is uncertainty regarding the effect of tranexamic acid on the incidence of thromboembolism in case of surgical bleeding [17, 18]. For this reason, evaluation of alternative agents is necessary. Another possible way of reducing blood loss is through the use of hypotensive epidural anesthesia [19]. Although this could reduce blood loss as well as rate of blood transfusion, hypotensive epidural anesthesia is contraindicated in patients with severe stenosis of the aortic or mitral valve, severe stenosis of the carotid or vertebral artery, and heart block [19, 20]. Furthermore, the use of cell salvage techniques could reduce both blood loss and the requirement for blood transfusion with low rates of adverse effects [19, 21]. However, the high cost of cell salvage techniques may limit their use [19, 21]. In addition to these methodologies, we used an agent administered topically with a low rate of adverse effects and contraindications. To the best of our knowledge, this study is one of the first to investigate the effect of the polysaccharide 4DryField*®* PH on blood loss after hip bipolar hemiarthroplasty. Since 4DryField® PH was administered only during wound closure, we expected to observe a relatively less significant effect on the intraoperative blood loss and a greater effect on the postoperative blood loss. We observed an immediate hemostatic effect of 4DryField® PH after local application, similar to the findings described by Korell et al.. [6, 22]. In addition, we were also able to show that the volume of postoperative hematoma was significantly reduced with the use of 4DryField® PH.

The total blood loss after surgery was calculated using Mercuriali’s formula [8, 9], based on the patient’s blood volume calculated using the Nadler formula [9, 10], which is advantageous since administered blood transfusions are taken into account. Therefore, direct comparison of the blood loss between patients receiving and those not receiving a blood transfusion was possible. We found that 4DryField® PH had no effect on total blood loss and drainage volume. On the other hand, a significant reduction on the volume of the postoperative hematoma was seen in the intervention group (an absolute reduction of 43.00 mL). Moreover, two patients of the control group needed surgical revision because of a large postoperative hematoma. Thus, the finding that total blood loss was unaffected can be explained by the fact that the reduction of hematoma size was relatively small in terms of volume, with the drainage volume being comparable between the two groups.

### Limitations

This study did not examine the effects of other factors, such as concurrent medications like anticoagulants, due to the small sample size. The findings of this pilot study are intended to serve as a basis for larger, future studies to generate evidence regarding various aspects of 4DryField® PH, including drug interactions with other medications, parallel use with tranexamic acid, and effects on arthroplasty outcomes. Moreover, the sample size was low according to the intended function as a pilot study. The effects evaluated in this study should be proven using a larger and multi-centric study protocol. Here, the larger applicator content of 9 g should also be investigated. In contrast to most studies, we used 3 redon drainages. Corresponding to our clinical standard, each compartment (submuscular, subfascial, and subcutaneous) was addressed by its own drainage. To fit the literature standard, we summed up the drainage volume, which could result in a bias.

Although the absolute frequencies of the anticoagulative agents were comparable, low-molecular-weight and unfractionated heparin were used, which could cause a bias.

The usage of the polysaccharide 4DryField*®* PH was described in literature only in cases of gynecology, visceral surgery, and in body-contouring surgery [23–25]. To the best of our knowledge there are no literature describing its usage in other disciplines, particularly in musculoskeletal surgery. Therefore, a comparison of our results to the literature findings is limited.

## Conclusions

In conclusion, this study is one of the first to investigate the effect of the polysaccharide 4DryField® PH on blood loss after hip bipolar hemiarthroplasty. The volume of the postoperative hematoma was reduced by 43.0 mL indicating the hemostatic effect of 4DryField® PH. However, this small reduction in volume was not reflected in total blood loss. In order to the low costs of 120 € for each application and introduce an easy-to-use agent, larger studies aimed at optimizing the administration algorithm and evaluating the use of this potential option for decreasing perioperative blood loss should be performed.

## Data Availability

The datasets generated and/or analyzed during the current study are not publicly available due data privacy rules but are available from the corresponding author on reasonable request.

## Abbreviations

SD: Standard deviation
IQR: Interquartile range
